# ﻿*Barbastellacaspica* (Chiroptera, Vespertilionidae) in China: first record and complete mitochondrial genome

**DOI:** 10.3897/zookeys.1228.137496

**Published:** 2025-02-18

**Authors:** Zhong-Yu Wang, Shamshidin Abduriyim

**Affiliations:** 1 College of Life Science, Shihezi University, Shihezi 832003, Xinjiang, China Shihezi University Shihezi China

**Keywords:** Echolocation calls, phylogenetic analysis, Xinjiang

## Abstract

The Caspian barbastelle, *Barbastellacaspica*, has spread widely in the Caspian region, Iran, and Central Asia; however, there is no evidence of its occurrence in China so far. During a field investigation, we collected a single specimen of *B.caspica* in China’s Xinjiang Uyghur Autonomous Region. At the same time, we obtained the free-flight echolocation calls of the bat. It omitted signals with start frequency of 33.15 ± 1.43 kHz, end frequency of 29.82 ± 0.40 kHz, frequency of most energy 31.48 ± 0.40 kHz, duration of 2.43 ± 0.24 ms, and a pulse interval of 246.57 ± 9.48 ms, which are probably type-I sounds emitted through the mouth. We also sequenced its entire mitochondrial genome to elucidate the genomic structure and its evolutionary relationships with closely related *Barbastella*. The mitochondrial genome of *B.caspica* spans 16,933 bp, comprising 13 protein-encoding genes, 22 transfer RNA genes, two ribosomal RNA genes, and a displacement loop/control region. Consistent with previous bat mitogenome reports, the majority of mitochondrial genes are encoded on the heavy chain. A phylogenetic analysis based on 13 protein-coding genes revealed that *Rhogeessa*, *Plecotus*, and *B.caspica* formed a clade within Vespertilionidae. *Barbastellacaspica* was found to be a sister species to *B.beijingensis* and *B.leucomelas* in phylogenetic trees using the cytochrome *b* and *ND1* gene sequences. This is the first report of the mitogenome of a member of the genus *Barbastella*, as well as the first record of the distribution of *B.caspica* in China and first documentation of its echolocation calls.

## ﻿Introduction

The *Barbastella* genus is widely distributed from Northeast Africa to across Eurasia to Taiwan and Japan. Currently, only six species are recognized: *B.barbastellus* Schreber, 1774, *B.beijingensis*Zhang et al., 2007, *B.caspica* Satunin, 1908, *B.darjelingensis* Hodogson, 1855, *B.leucomelas* Cretzschmar, 1826, and *B.pacifica*Kruskop et al., 2019 (https://www.checklistbank.org/). In China, distributional records exist only for *B.beijingensis* and *B.darjelingensis* (http://www.sp2000.org.cn/).

The Caspian barbastelle, *B.caspica*, primarily inhabits drier habitats and is occasionally found in caves, crevices, and mines. Its main distribution encompasses northern Iran, the Caucasus region (Armenia, Azerbaijan, and Dagestan in Russia), Uzbekistan, and Tajikistan (Kruskop 2015). Research on this species is relatively limited, with a few studies focusing on taxonomic status and distribution (Kruskop et al. 2019). Furthermore, genomic studies on species of *Barbastella* have been lacking, and the phylogenetic position of this genus within the family Vespertilionidae has not been explored.

In this study, we used mist nets to capture and ultrasound recording equipment to record *B.caspica* echolocation calls. Furthermore, we conducted a comprehensive assembly and analysis of the complete mitochondrial genome of *B.caspica*, thus establishing the first genomic resource of *Barbastella*. Specifically, we analyzed the nucleotide composition of the entire mitochondrial DNA molecule, investigated the codon usage patterns and selective constraints of protein-coding genes (PCGs), and described the secondary structure of each identified tRNA gene. Finally, based on mitochondrial PCGs, cytochrome *b* (*Cytb*), and *ND1* sequences, we examined the phylogenetic position of *Barbastella* among other representative species of Vespertilionidae and of *B.caspica* within its genus. On the one hand, the complete assembly of mitochondrial genome markers was a significant step toward advancing our understanding of the genomic evolutionary biology and systematics of *Barbastella* species. On the other hand, this study also reported the first documentation of this species in China and the features of echolocation calls during flight.

## ﻿Materials and methods

**Figure 1.**
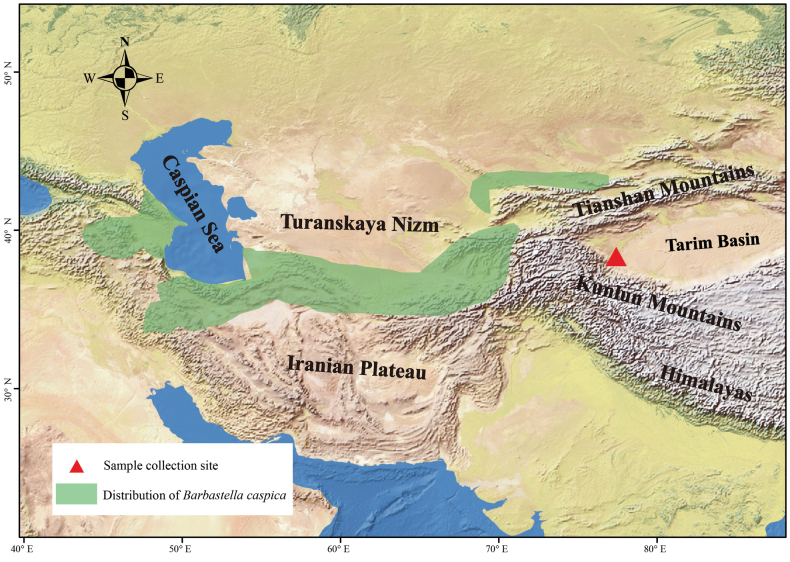
Map of Central Asia showing the geographic range of *Barbastellacaspica* (green) and sampling site (red triangle) in southern Xinjiang, China.

A bat individual was captured using mist nets during a survey of chiropteran resources in Yarkand County (37°54'24.75"N, 76°47'2.86"E), Xinjiang Uygur Autonomous Region of China, in July 2023 (Fig. 1). The specimen (SC230705005) is currently stored at the College of Life Sciences, Shihezi University. Morphological identification revealed that the bat had short, wide ears with the front ends of both ears connected, indicating that it belongs to a species of barbastelles bat, *Barbastella* genus (https://www.checklistbank.org/). A Song Meter SM4BAT FS ultrasonic recording device (Wildlife Acoustics, USA) was placed next to the mist net to record bat echolocation calls. Subsequently, the recorded echolocation sound waves were analyzed using sound analysis software (Kaleidoscope v. 5.4.8).

In the laboratory, total genomic DNAs were extracted from muscle tissues using the Tiancheng Genomic DNA Extraction Kit (Tiangen Biotech, Beijing, China). The mitochondrial genome of *B.caspica* was amplified using PCR with 11 pairs of custom-designed primers (Suppl. material 1). Products that met quality-control criteria were purified and commercially sequenced. Sequencing data were processed and assembled using SeqMan software (Tamura et al. 2013). The annotation of the mitochondrial genome was performed using the GeSeq organelle genome annotation server (Tillich et al. 2017) (https://chlorobox.mpimp-golm.mpg.de/geseq.html). Annotation refinement and adjustment of start/stop codons were performed using MEGA X (Kumar et al. 2018). The finalized mitochondrial sequence has been deposited in NCBI GenBank under accession number PP963575.

## ﻿Results and discussion

**Figure 2.**
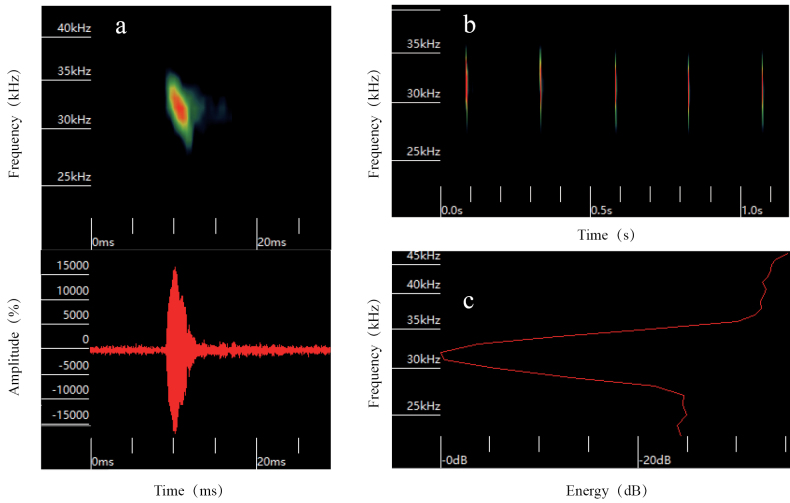
Echolocation calls features of *B.caspica* in free flight conditions: the spectrogram and waveform with time unit in milliseconds (**a**), the spectrogram with a time unit of seconds (**b**) and the energy spectrum (**c**).

The echolocation call of *Barbastellacaspica* is characterized by frequency modulation (FM) (Fig. 2a, b). In free-flight outdoor conditions, the pulses are composed of a single harmonic. The peak frequency is notably low, with the highest energy peak occurring at 31.48 ± 0.40 kHz (Fig. 2c). The frequency bandwidth is narrow, measuring only 5.79 ± 1.04 kHz. The initial frequency is at 33.15 ± 1.43 kHz and the final frequency is at 29.82 ± 0.40 kHz. The pulse duration is relatively short, approximately 2.43 ± 0.24 ms, with an interpulse interval of 246.57 ± 9.48 ms (Table 1). These characteristics closely resemble the sound waves emitted by other species of *Barbastella* while foraging (Zhang et al. 2007) and were similar to the type-I sounds of *Barbastella* species (Denzinger et al. 2001). However, considering that certain species of horseshoe bat consistently emit two different types of sound waves during foraging (Seibert et al. 2015), it is possible that our sound-wave detector failed to capture type-II sounds. Alternatively, it is likely that *B.caspica* does not produce this particular sound during foraging or that the frequency of the emitted sound waves is lower than in other *Barbastella* species. These possibilities should be confirmed in future studies.

**Table 1.** Echolocation calls features of *Barbastellacaspica* in free-flight conditions.

Items	Range	Mean ± SD
Initial frequency (kHz)	29.97–34.63	33.15 ± 1.43
Terminate frequency (kHz)	28.99–30.19	29.82 ± 0.40
Frequency bandwidth (kHz)	4.02–7.27	5.79 ± 1.04
Main frequency (kHz)	31.07–31.96	31.48 ± 0.40
Duration time (ms)	2.05–2.74	2.43 ± 0.24
Interval time (ms)	232.29–266.43	246.57 ± 9.48

**Figure 3.**
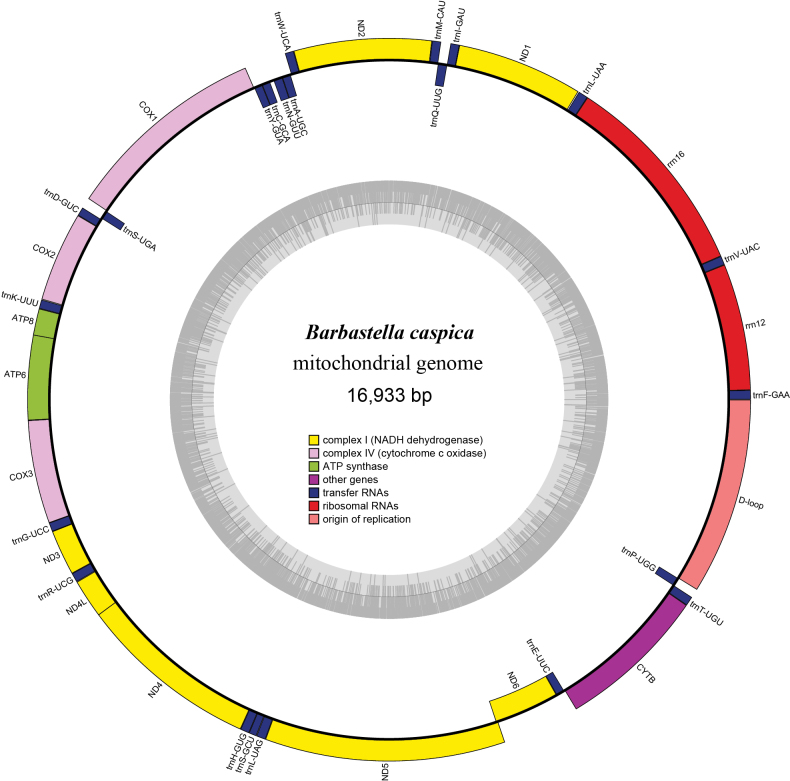
Mitochondrial genome map of *B.caspica*. The mitochondrial DNA of *B.caspica* is 16,933 base pairs long, consisting of different segments: 22 blue segments representing tRNA coding regions, 2 red segments corresponding to 12SrRNA and 16SrRNA, 7 yellow segments for *ND1*, *ND2*, *ND3*, *ND4L*, *ND4*, *ND5*, and *ND6*, 3 pink segments for *COX1*, *COX2*, and *COX3*, 1 purple segment for the *Cytb* gene, and 1 light red segment for the D-loop region.

The mitochondrial genome of *B.caspica* is a circular DNA molecule with a length of 16,933 base pairs (Fig. 3). The genome encompasses a total of 37 genes, consisting of 13 PCGs, 22 transfer RNA genes (tRNAs), two ribosomal RNA genes (rRNAs), and one D-loop region. The size and organization of these mitochondrial genes (Table 2) are consistent with previous reports of other vespertilionid species (Guo et al. 2021; Martínez-Cárdenas et al. 2024; Valencia M. et al. 2024). Among the 13 PCGs (11,408 bp), they exhibit similarities with other species of Vespertilionidae, such as being located on the heavy strand except for *ND6* (Martínez-Cárdenas et al. 2024; Valencia M. et al. 2024). The average A+T content of PCGs in mitochondria is 59.92%, ranging from 56.31% (*COX1*) to 64.73% (*ATP8*), which is higher than the G+C content (40.08%) of the 13 PCGs. Furthermore, they show similar negative AT skew and CG skew, as well as a high A+G content (60.03%) (Suppl. material 2) (Guo et al. 2021; Martínez-Cárdenas et al. 2024; Valencia M. et al. 2024). All PCGs start with ATG or ATA codons and terminate with TAA or truncated T residues, except for the *Cytb* gene, which terminates with AGA (Table 2).

**Table 2.** Composition and organization of the mitochondrial genome of *Barbastellacaspica*.

Gene	Strand	Location	Size(bp)	Start Codon	Stop Codon	Anticodon	Continuity
tRNA-Phe	H	1–72	72	–	–	GAA	0
12S Rrna	H	72–1031	960	–	–	–	−1
tRNA-Val	H	1032–1100	69	–	–	TAC	0
16S rRNA	H	1101–2668	1569	–	–	–	0
tRNA-Leu2	H	2669–2743	75	–	–	TAA	−1
ND1	H	2749–3705	957	ATG	TAA	–	5
tRNA-Ile	H	3705–3772	68	–	–	GAT	−1
tRNA-Gln	L	3770–3843	74	–	–	TTG	−3
tRNA-Met	H	3844–3911	68	–	–	CAT	0
ND2	H	3912–4953	1042	ATA	T––	–	0
tRNA-Trp	H	4954–5020	67	–	–	TCA	0
tRNA-Ala	L	5028–5095	68	–	–	TGC	7
tRNA-Asn	L	5096–5168	73	–	–	GTT	0
tRNA-Cys	L	5200–5266	67	–	–	GCA	31
tRNA-Tyr	L	5267–5332	66	–	–	GTA	0
COX1	H	5334–6878	1545	ATG	TAA	–	1
tRNA-Ser2	L	6882–6950	69	–	–	TGA	3
tRNA-Asp	H	6958–7024	67	–	–	GTC	7
COX2	H	7025–7708	684	ATG	TAA	–	0
tRNA-Lys	H	7711–7779	69	–	–	TTT	2
ATP8	H	7780–7983	204	ATG	TAA	–	0
ATP6	H	7941–8621	681	ATG	TAA	–	−43
COX3	H	8621–9404	784	ATG	T––	–	−1
tRNA-Gly	H	9404–9472	69	–	–	TCC	−1
ND3	H	9472–9818	347	ATA	TA–	–	−1
tRNA-Arg	H	9819–9889	71	–	–	TCG	0
ND4L	H	9891–10187	297	ATG	TAA	–	1
ND4	H	10181–11558	1378	ATG	T––	–	−7
tRNA-His	H	11559–11627	69	–	–	GTG	0
tRNA-Ser1	H	11628–11686	59	–	–	GCT	0
tRNA-Leu1	H	11688–11758	71	–	–	TAG	1
ND5	H	11759–13579	1821	ATA	TAA	–	0
ND6	L	13563–14090	528	ATG	TAA	–	−17
tRNA-Glu	L	14091–14158	68	–	–	TTC	0
Cytb	H	14164–15303	1140	ATG	AGA	–	5
tRNA-Thr	H	15304–15375	72	–	–	TGT	0
tRNA-Pro	L	15373–15441	69	–	–	TGG	−3
D-loop	H	15442–16933	1492	–	–	–	0

Suppl. material 3 shows the codon counts and RSCU values of *B.caspica*. The 33 codons are used more frequently (RSCU > 1, Suppl. material 4). The codons AAU-Asn (158), ACA-Thr (133), CCA-Pro (130), ACU-Thr (121), and CUA-Leu (118) are the most frequently used. There are 22 typical tRNA genes, ranging in length from 59 bp (tRNA-Ser1) to 75 bp (tRNA-Leu2). Eight of these genes are located on the L strand, while 14 are on the H strand. In total, they span 1520 bp. Except for tRNA-Ser (Table 2, Suppl. material 5), all these tRNA molecules have the classical cloverleaf structure. This phenomenon has been mentioned in previous studies and is common among metazoans (Vivas-Toro et al. 2021; Basaldúa et al. 2023). However, more research is needed to determine the functionality of these features in *B.caspica*.

**Figure 4.**
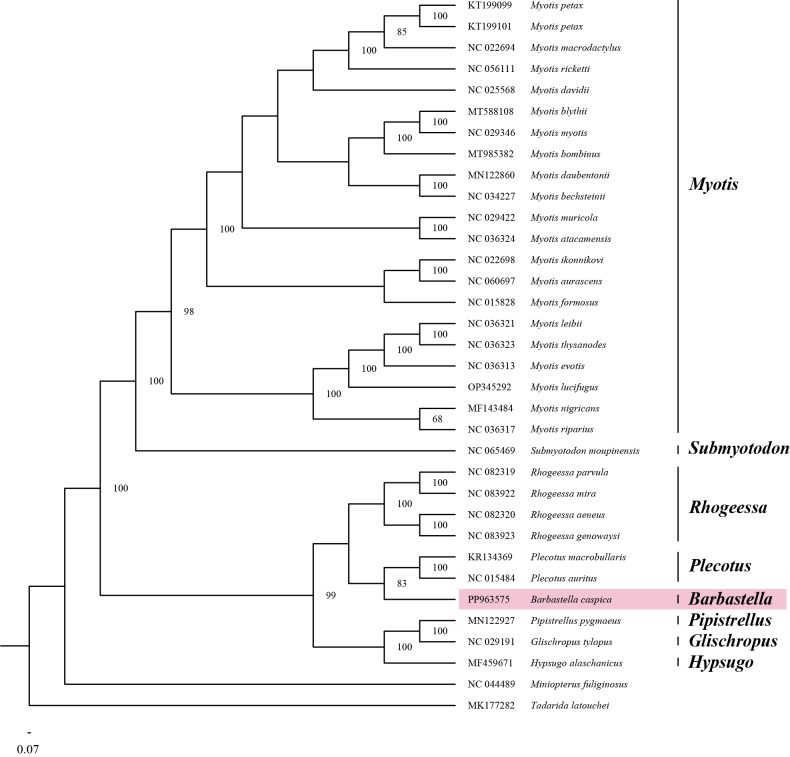
The phylogenetic relationships of the Vespertilionidae based on 13 protein-coding genes using the maximum-likelihood method with 1,000 bootstrap replicates. *Tadaridalatouchei* and *Miniopterusfuliginosus* were designated as outgroups. Nodes with support values ≥ 80 are indicated.

Based on 13 PCG sequences, we successfully constructed the phylogenetic topology of 31 species from the vespertilionid subfamilies Myotinae and Vespertilioninae. Consequently, *Rhogeessa*, *Plecotus*, *Pipistrellus*, *Glischropus*, *Hypsugo*, and *Barbastella* formed the subfamily Vespertilioninae, with *Barbastella* being a sister genus to *Plecotus* (Fig. 4). Consistent with previous results based on the *COI* gene (Chakravarty et al. 2020), we found that *Plecotus* and *Barbastella* belonged to the same tribe, Plecotini, which also includes four other genera (Wilson and Mittermeier 2019; https://www.checklistbank.org/), implying that more genome-based phylogeny is required to understand the intergeneric evolutionary relationships within the Plecotini.

**Figure 5.**
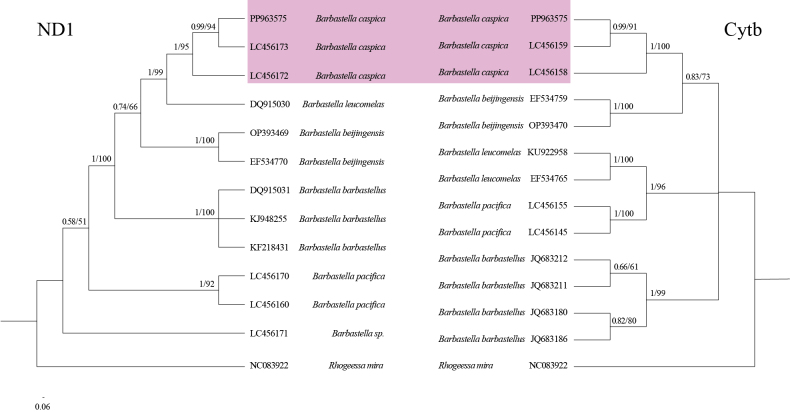
The Bayesian analyses of phylogenetic relationships of members of the *Barbastella* genus based on 806 bp *ND1* (left) and 1140 bp *Cytb* (right) sequences using Bayesian-inference (BI) and maximum-likelihood (ML) methods. *Rhogeessamira* is used as the root, and nodes with support values of ≥ 0.7 (BI) and 80 (ML) are labeled.

Phylogenetic trees were constructed to elucidate the evolutionary relationship of *B.caspica* with other species of vespertilionids, based on the *Cytb* and *ND1* genes along with all PCGs. Within *Barbastella*, *B.caspica* is identified as a distinct species. However, differential topological structures were observed in the phylogenetic trees constructed based on the *ND1* and *Cytb* genes (Fig. 5). Namely, *B.caspica* was a sister species to *B.leucomelas* in the *ND1* phylogenetic tree, but sister to *B.beijingensis* in the *Cytb* phylogenetic tree.

The pairwise distances (Table 3) shows that the smallest genetic distances (3.7% based on the *ND1* gene) are between *B.caspica* and *B.leucomelas*. However, the direct pairwise distance between the two species based on the *Cytb* gene is 13.4%, which is consistent with the results of the phylogenetic tree.

**Table 3.** ML distances (above the diagonal) and *p*-distances (below the diagonal) (in %) for *ND1* and *Cytb* sequences of *Barbastellacaspica.*

Species	*B.caspica*	*B.leucomelas*	*B.beijingensis*	*B.barbastellus*	*B.pacifica*
***B.caspica***	–	5.0 / 15.4	12.8 / 13.6	14.1 / 16.1	16.9 / 16.0
***B.leucomelas***	3.7 / 13.4	–	13.2 / 15.3	14.7 / 16.6	18.2 / 14.9
***B.beijingensis***	9.4 / 12.0	9.6 / 18.0	–	15.4 / 18.4	17.1 / 17.6
***B.barbastellus***	10.6 / 13.9	10.8 / 14.2	11.1 / 15.7	–	17.9 / 15.2
***B.pacifica***	12.6 / 13.8	13.5 / 13.1	12.6 / 14.9	13.3 / 17.9	–

The systematic construction of the *ND1* phylogenetic tree, as well as the *ND1* genetic distances within *Barbastella*, consistently indicate a close genetic relationship between *B.caspica* and *B.leucomelas*, which agrees with the results of Smirnov et al. (2020). In contrast, the findings of the *Cytb* analyses are conflicting (Fig. 5, Table 3). Phylogenetic tree inconsistencies are common among mammals, especially due to important evolutionary events (Rokas and Chatzimanolis 2008). These discrepancies can be attributed to factors such as inadequate gene sampling, hybridization events, gene introgression, or horizontal transfer. Although these findings provide enough evidence to consider *B.caspica* as an independent species (Fig. 5, Table 3), our understanding of its evolutionary relationship with other *Barbastella* species remains limited. Furthermore, the Central Asian species is named *B.walteri* (Smirnov et al., 2021). Hence, to obtain more comprehensive information, it is necessary to explore the genomic aspects of all species rather than confining our study solely to partial genes.

Previous reports have indicated that *B.caspica* is distributed from the Caucasus region through Iran to Tajikistan (Kruskop 2015), excluding China. This report has expanded our understanding of the geographic distribution of *B.caspica*. Combining these findings with previous research, we infer that the eastern edge of the *B.caspica* distribution extends to Xinjiang, China. Before this discovery, only two species of barbastelles (*B.darjelingensis* and *B.beijingensis*) had been documented in China, and *B.darjelingensis* was found exclusively in Xinjiang. Therefore, our report adds an additional species of barbastelle bats to the Chinese biodiversity.

## ﻿Conclusions

This study highlights the presence of *B.caspica* in Xinjiang, China, for the first time and presents the first complete assembly of the mitochondrial genome, providing valuable genetic resources for investigating inter- and intraspecific evolutionary relationships. In addition, we describe for the first time free-flight echolocation calls, possibly of type-I sounds omitted through the mouth. Taking the collection site of our specimen of *B.caspica* into account, it is necessary to conduct further ecological and genetic studies at the population level on a whole distributional scale.

## ﻿Additional information

### Author ORCIDs

Zhong-Yu Wang https://orcid.org/0009-0002-6277-942X

Shamshidin Abduriyim https://orcid.org/0000-0002-7038-077X

### Data availability

The mtDNA sequences we obtained have been deposited in the NCBI GenBank databases under accession numbers PP963575.

## Supplementary materials

Supplementary material 1 PCR primers designed for mitochondrial genome analysis of *B.caspica* Data type docx https://binary.pensoft.net/file/1254410This dataset is made available under the Open Database License (http://opendatacommons.org/licenses/odbl/1.0/). The Open Database License (ODbL) is a license agreement intended to allow users to freely share, modify, and use this Dataset while maintaining this same freedom for others, provided that the original source and author(s) are credited.Zhong-Yu Wang , Shamshidin Abduriyim

Supplementary material 2 Base composition of the mitogenomes of *B.caspica* Data type docx https://binary.pensoft.net/file/1254411This dataset is made available under the Open Database License (http://opendatacommons.org/licenses/odbl/1.0/). The Open Database License (ODbL) is a license agreement intended to allow users to freely share, modify, and use this Dataset while maintaining this same freedom for others, provided that the original source and author(s) are credited.Zhong-Yu Wang , Shamshidin Abduriyim

Supplementary material 3 RSCU values of protein-coding genes in mitochondrial genome of *B.caspica* Data type pdf https://binary.pensoft.net/file/1254412This dataset is made available under the Open Database License (http://opendatacommons.org/licenses/odbl/1.0/). The Open Database License (ODbL) is a license agreement intended to allow users to freely share, modify, and use this Dataset while maintaining this same freedom for others, provided that the original source and author(s) are credited.Zhong-Yu Wang , Shamshidin Abduriyim

Supplementary material 4 Frequency and RSCU values of codon in protein coding genes in the mitogenome of *B.caspica* Data type docx https://binary.pensoft.net/file/1254413This dataset is made available under the Open Database License (http://opendatacommons.org/licenses/odbl/1.0/). The Open Database License (ODbL) is a license agreement intended to allow users to freely share, modify, and use this Dataset while maintaining this same freedom for others, provided that the original source and author(s) are credited.Zhong-Yu Wang , Shamshidin Abduriyim

Supplementary material 5 The secondary structure of tRNA gene Data type pdf https://binary.pensoft.net/file/1254414This dataset is made available under the Open Database License (http://opendatacommons.org/licenses/odbl/1.0/). The Open Database License (ODbL) is a license agreement intended to allow users to freely share, modify, and use this Dataset while maintaining this same freedom for others, provided that the original source and author(s) are credited.Zhong-Yu Wang , Shamshidin Abduriyim
