# Supplementary material for: ﻿Barbastellacaspica (Chiroptera, Vespertilionidae) in China: first record and complete mitochondrial genome
Source: Zookeys. 2025 Feb 18;1228:115–26. doi: 10.3897/zookeys.1228.137496 (PMC11862895; doi:10.3897/zookeys.1228.137496)
Supplement: Supplementary material 1 — PCR primers designed for mitochondrial genome analysis of B.caspica [file zookeys-1228-115_article-137496__-s001.docx]

**Suppl. material 1.** PCR primers designed for mitochondrial genome analysis of *B. caspica*

| Primers | Upstream sequence (5'-3') | Downstream sequence (5'-3') | Product length // bp |
| --- | --- | --- | --- |
| 1 | TTACACATGCAAGAATCC | CTTAAGTTTTATGCAATTAC | 2595 |
| 2 | TACGACCTCGATGTTGGA | GTTGCTGCTTCTGTGGAT | 1670 |
| 3 | GGTCATTCTCATTACCAACAC | CGAGTAGGCATTAGACTGTAA | 2101 |
| 4 | GGCAGGATTGAAGCTGCTTCT | AATAAATTTTCGTTCATTT | 2750 |
| 5 | TCCAGACCAGAAGCCTTC | GCGGTTACGACTACATTGT | 545 |
| 6 | CACAGACTATGAGGACTTACT | CACATTCGTATGGACTTGAT | 2236 |
| 7 | CCTTTTAAGTTAGAGA | TGTGGAGTTAATTCATA | 2539 |
| 7 | TATTTGTACTAATTAC | AGGCGTTTGTATAAGACGTGT | 2978 |
| 8 | GCCTCCATCTATGACTACC | TTGAAGAATGCGTGAGTG | 1954 |
| 9 | ATTAAAAAAATTGTAGC | TTGGGTTATTAGATCC | 2480 |
| 10 | CCTTGTTGAATGAATTTGAGG | GGGCCTTTGACGGCCAATG | 1805 |
| 11 | CATCTGGTTCTTACTTCAGGC | GCTGGCACGAGATTTACCAGT | 1000 |
