# Supplementary material for: ﻿Barbastellacaspica (Chiroptera, Vespertilionidae) in China: first record and complete mitochondrial genome
Source: Zookeys. 2025 Feb 18;1228:115–26. doi: 10.3897/zookeys.1228.137496 (PMC11862895; doi:10.3897/zookeys.1228.137496)
Supplement: Supplementary material 2 — Base composition of the mitogenomes of B.caspica [file zookeys-1228-115_article-137496__-s002.docx]

**Suppl. material 2.** Base composition of the mitogenomes of *B. caspica*

| **Gene** | **Base composition (%)** | | | | **A+T(%)** | **ATskew** | **GCskew** |
| --- | --- | --- | --- | --- | --- | --- | --- |
|  | **A** | **T** | **C** | **G** |  |  |  |
| 12s ribosomal RNA | 36.15 | 21.25 | 23.54 | 19.06 | 57.40 | 0.260 | -0.105 |
| 16s ribosomal RNA | 37.69 | 22.19 | 22.83 | 17.28 | 59.89 | 0.259 | -0.138 |
| ND1 | 31.97 | 26.33 | 28.94 | 12.75 | 58.31 | 0.097 | -0.388 |
| ND2 | 37.33 | 24.57 | 28.69 | 9.40 | 61.90 | 0.206 | -0.506 |
| COX1 | 26.80 | 29.51 | 26.08 | 17.61 | 56.31 | -0.048 | -0.194 |
| COX2 | 32.31 | 25.73 | 27.92 | 14.04 | 58.04 | 0.113 | -0.331 |
| ATP8 | 39.22 | 24.51 | 28.43 | 7.84 | 63.73 | 0.231 | -0.568 |
| ATP6 | 31.57 | 27.17 | 29.22 | 12.04 | 58.74 | 0.075 | -0.416 |
| COX3 | 28.44 | 28.19 | 28.57 | 14.80 | 56.63 | 0.005 | -0.318 |
| ND3 | 31.70 | 31.41 | 25.07 | 11.82 | 63.11 | 0.005 | -0.359 |
| ND4L | 32.32 | 26.94 | 30.30 | 10.44 | 59.26 | 0.091 | -0.488 |
| ND4 | 33.02 | 27.94 | 28.01 | 11.03 | 60.96 | 0.083 | -0.435 |
| ND5 | 33.39 | 27.57 | 28.17 | 10.87 | 60.96 | 0.095 | -0.443 |
| ND6 | 18.37 | 45.27 | 6.44 | 29.92 | 63.64 | -0.423 | 0.646 |
| CYTB | 30.44 | 26.93 | 29.47 | 13.16 | 57.37 | 0.061 | -0.383 |
| D-loop | 36.80 | 27.95 | 22.25 | 13.00 | 64.75 | 0.137 | -0.262 |
