# Supplementary figures and images for: ﻿Barbastellacaspica (Chiroptera, Vespertilionidae) in China: first record and complete mitochondrial genome
Source: Zookeys. 2025 Feb 18;1228:115–26. doi: 10.3897/zookeys.1228.137496 (PMC11862895; doi:10.3897/zookeys.1228.137496)

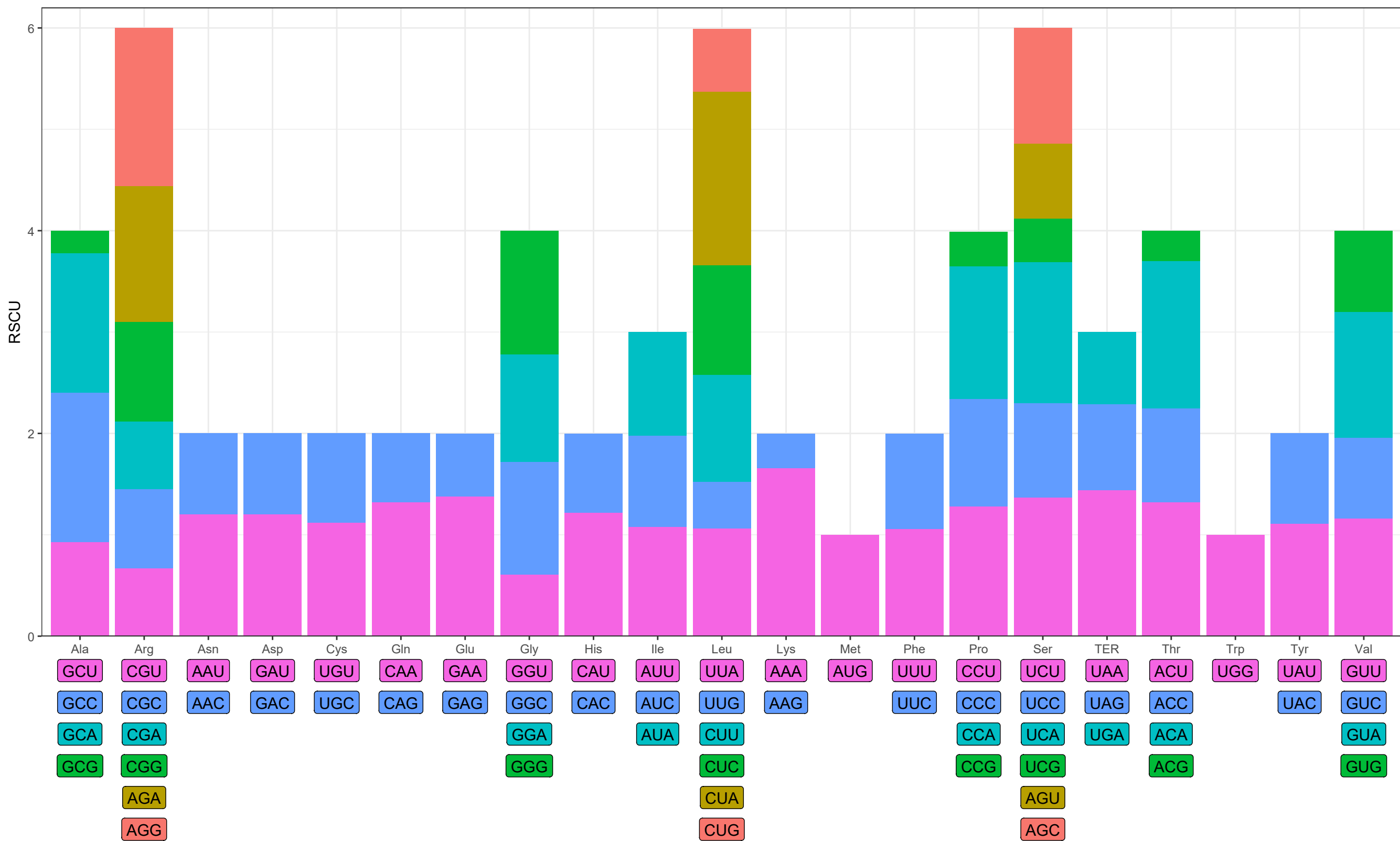

Supplement: Supplementary material 3 — RSCU values of protein-coding genes in mitochondrial genome of B.caspica [file zookeys-1228-115_article-137496__-s003.pdf]

*Arg*

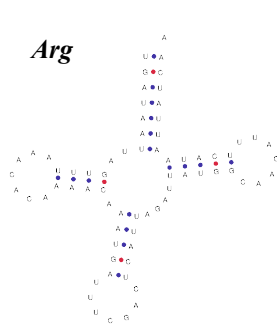

*Gly*

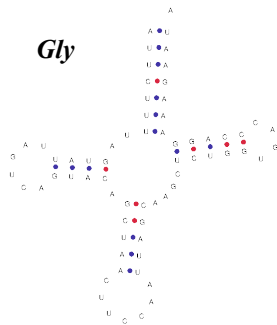

*Lys*

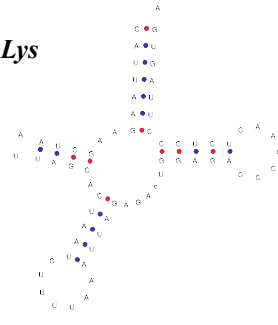

*Leu1*

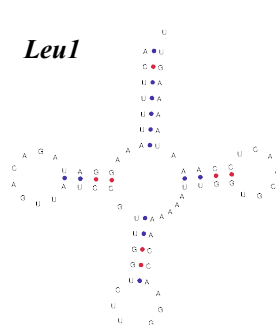

*His*

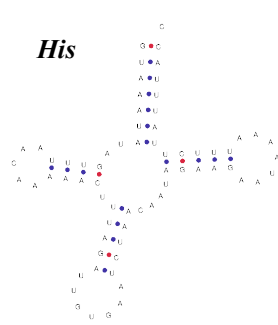

*Asp*

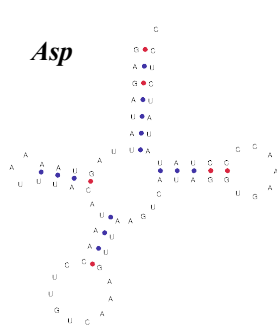

*Thr*

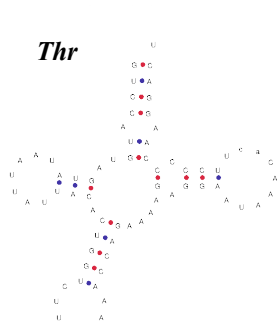

*Pro*

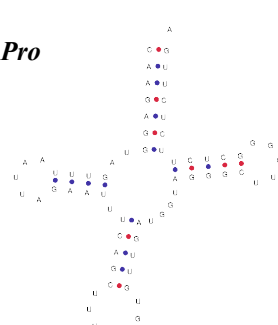

*Ser2*

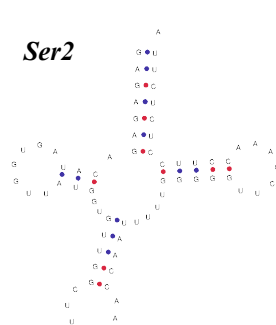

*Tyr*

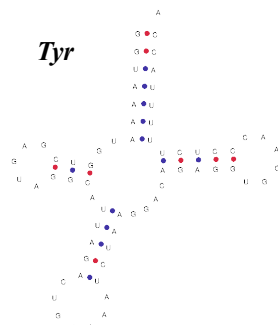

*Met*

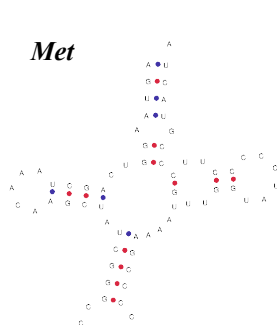

*Cys*

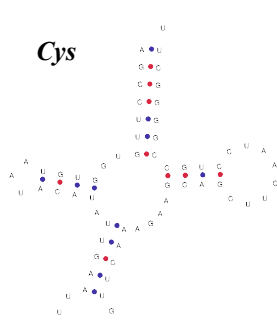

*Gln*

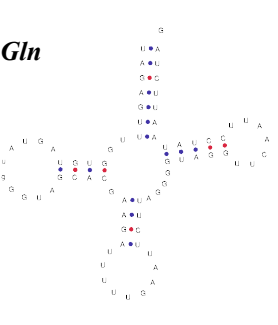

*Asn*

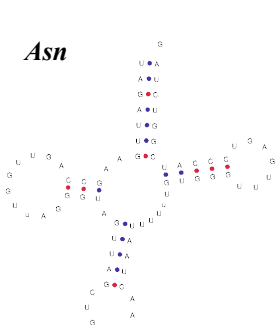

*Ala*

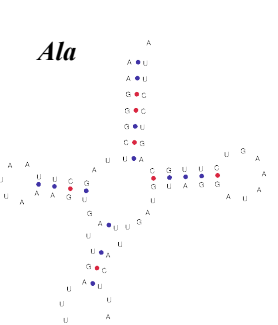

*Trp*

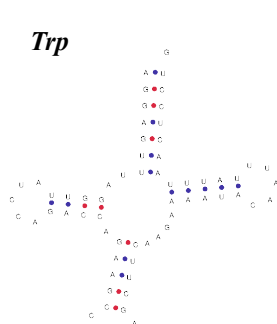

*Glu*

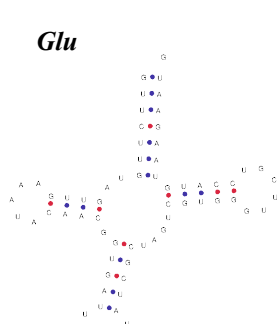

*Ser1*

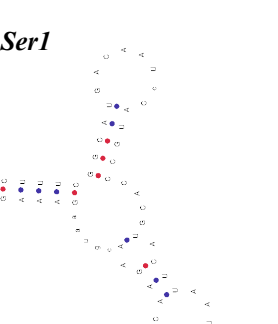

*Ile*

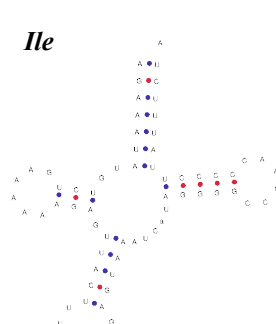

*Phe*

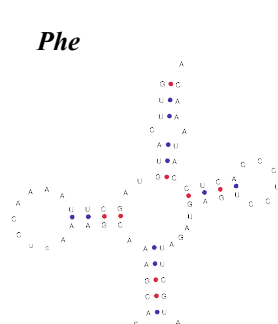

*Val*

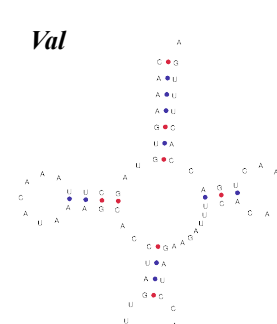

*Leu2*

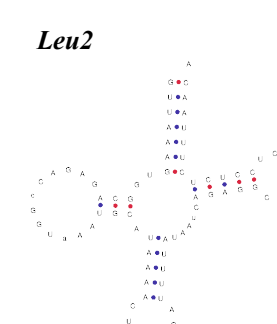

Supplement: Supplementary material 5 — The secondary structure of tRNA gene [file zookeys-1228-115_article-137496__-s005.pdf]
