# Supplementary material for: ﻿Barbastellacaspica (Chiroptera, Vespertilionidae) in China: first record and complete mitochondrial genome
Source: Zookeys. 2025 Feb 18;1228:115–26. doi: 10.3897/zookeys.1228.137496 (PMC11862895; doi:10.3897/zookeys.1228.137496)
Supplement: Supplementary material 4 — Frequency and RSCU values of codon in protein coding genes in the mitogenome of B.caspica [file zookeys-1228-115_article-137496__-s004.docx]

**Suppl. material 4.** Frequency and RSCU values of codon in protein coding genes in the mitogenome of *B. caspica*.

| AA | Codon | Count | RSCU |  | AA | Codon | Count | RSCU |
| --- | --- | --- | --- | --- | --- | --- | --- | --- |
| Ala | GCU | 29 | 0.93 |  | Met | AUG | 46 | 1 |
|  | GCC | 46 | 1.47 |  | Leu | UUA | 73 | 1.06 |
|  | GCA | 43 | 1.38 |  |  | UUG | 32 | 0.46 |
|  | GCG | 7 | 0.22 |  |  | CUU | 73 | 1.06 |
| Arg | CGU | 24 | 0.67 |  |  | CUC | 74 | 1.08 |
|  | CGC | 28 | 0.78 |  |  | CUA | 118 | 1.71 |
|  | CGA | 24 | 0.67 |  |  | CUG | 43 | 0.62 |
|  | CGG | 35 | 0.98 |  | Pro | CCU | 127 | 1.28 |
|  | AGA | 48 | 1.34 |  |  | CCC | 105 | 1.06 |
|  | AGG | 56 | 1.56 |  |  | CCA | 130 | 1.31 |
| Asn | AAU | 158 | 1.2 |  |  | CCG | 34 | 0.34 |
|  | AAC | 106 | 0.8 |  | Ser | UCU | 84 | 1.37 |
| Asp | GAU | 39 | 1.2 |  |  | UCC | 57 | 0.93 |
|  | GAC | 26 | 0.8 |  |  | UCA | 85 | 1.39 |
| Cys | UGU | 29 | 1.12 |  |  | UCG | 26 | 0.43 |
|  | UGC | 23 | 0.88 |  |  | AGU | 45 | 0.74 |
| Gln | CAA | 75 | 1.32 |  |  | AGC | 70 | 1.14 |
|  | CAG | 39 | 0.68 |  | Ter | UAA | 102 | 1.44 |
| Gly | GGU | 12 | 0.61 |  |  | UAG | 60 | 0.85 |
|  | GGC | 22 | 1.11 |  |  | UGA | 50 | 0.71 |
|  | GGA | 21 | 1.06 |  | Thr | ACU | 121 | 1.32 |
|  | GGG | 24 | 1.22 |  |  | ACC | 86 | 0.93 |
| His | CAU | 112 | 1.22 |  |  | ACA | 133 | 1.45 |
|  | CAC | 71 | 0.78 |  |  | ACG | 28 | 0.3 |
| Ile | AUU | 109 | 1.08 |  | Trp | UGG | 14 | 1 |
|  | AUC | 91 | 0.9 |  | Tyr | UAU | 115 | 1.11 |
|  | AUA | 103 | 1.02 |  |  | UAC | 92 | 0.89 |
| Lys | AAA | 112 | 1.66 |  | Val | GUU | 16 | 1.16 |
|  | AAG | 23 | 0.34 |  |  | GUC | 11 | 0.8 |
| Phe | UUU | 68 | 1.06 |  |  | GUA | 17 | 1.24 |
|  | UUC | 60 | 0.94 |  |  | GUG | 11 | 0.8 |
